# Determination of Four PAHs and Formaldehyde in Traditionally Smoked Chicken Products

**DOI:** 10.3390/molecules28135143

**Published:** 2023-06-30

**Authors:** Xinxuan Li, Yaohua Gao, Pinghua Deng, Xiaopu Ren, Shuang Teng

**Affiliations:** 1 College of Food Science and Technology, Nanjing Agricultural University, Nanjing 210095, China; 2 National Center of Meat Quality and Safety Control, Nanjing Agricultural University, Nanjing 210095, China; 3 Rizhao Customs, Rizhao 276826, China; 4 College of Life Science, Tarim University, Alar 843300, China

**Keywords:** traditionally smoked chicken products, polycyclic aromatic hydrocarbons, formaldehyde, safety

## Abstract

The present study was conducted to analyze the level of four priority polycyclic aromatic hydrocarbons (PAHs), including benzo[a]pyrene (BaP), chrysene (Chr), benzo[a]anthracene (BaA), and benzo[b]fluoranthene (BbF), in traditionally smoked chicken products marketed in China. The results show that the amount of ƩPAH4 (the sum of four different PAHs: BaP, Chr, BaA, and BbF) was 30.43–225.17 and 18.75–129.54 µg/kg in the skin and meat of smoked chicken products, respectively. The content of ƩPAH4 in the smoked skin was significantly higher as compared to the smoked meat (*p* < 0.05). The calculation of MOE (margin of exposure) results suggested the possibilities of ingestion risk associated with the consumption of smoked chicken skin. Furthermore, the formaldehyde content in the skin of smoked chicken was 2.17–6.84 mg/kg and 0.86–2.95 mg/kg in the smoked meat. These results indicate that optimization or alternative methods for food processing should be developed to reduce the high level of harmful substances formed during processing to ensure the safety of smoked chicken products. Moreover, along with harmful substances, the moisture content and color of traditionally smoked chicken were analyzed to provide a practical reference for healthy, safe and green processing technology for smoked chicken.

## 1. Introduction

Food safety issues have received considerable attention in recent years [1]. Smoked food products produced around the world are highly popular among consumers. However, the food safety concerns associated with such products have also attracted several researchers, because during the formation of the unique smoky flavors and colors of smoked meat products, a large number of harmful substances [2], such as polycyclic aromatic hydrocarbons (PAHs) [3,4] and formaldehyde [5], are generally formed.

Polycyclic aromatic hydrocarbons (PAHs) are organic compounds containing two or more fused aromatic rings of carbon and hydrogen atoms [6]. It is stated that about 660 compounds belong to the PAH group. PAHs are mainly produced by the incomplete combustion of organic matter [7]. The incomplete combustion of wood generates a large number of PAHs during the smoking process [8,9]. The formation of PAHs is affected by factors such as smoking temperature, smoking time, smoking method, and type of smoking material [10]. PAHs are recognized worldwide as highly toxic pollutants that are teratogenic, carcinogenic, and mutagenic [11,12]. They can enter the human body via the respiratory tract, skin, and digestive system and bind to proteins and nucleic acids. This leads to cell mutations and eventually results in the formation of malignant tumors, which pose serious threats to human health [13,14,15]. Currently, a total of 16 PAHs have been designated High-Priority Pollutants by the United States Environmental Protection Agency (US EPA), which include naphthalene (NA), acenaphthylene (ACY), acenaphthene (ACE), fluorene (FLU), phenanthrene (PHEN), anthracene (ANTH), fluoranthene (FLTH), pyrene (PYR), benzo [a]anthracene (BaA), chrysene (Chr), benzo[b]fluoranthene (BbF), benzo[k]fluoranthene (BkF), benzo[a]pyrene (BaP), benzo[g,h,i]perylene (BghiP), indeno[1,2,3-c,d]pyrene (IncdP), and dibenz[a,h]anthracene (DbahA). The International Agency for Research on Cancer (IARC) classifies BaP as a human carcinogen (group 1), whereas BaA, BbF, and Chr are possibly carcinogenic to humans (group 2B) [16]. Therefore, BaP is suggested as a marker that can be used to assess the effect of carcinogenic PAHs in food [17]. The European Food Safety Agency (EFSA) concluded that PAH8 (including BaA, BaP, BbF, BkF, BghiP, Chr, DbahA, and IncdP) and PAH4 (including BaA, BaP, Chr, and BbF) should be used as a marker of carcinogenic effects and genotoxicity of PAHs in food. However, PAH8 does not provide a significant amount of extra information compared to PAH4 [18,19]. It is well known from the literature that the level of PAH4, a combination of four major PAHs (BaP, Chr, BaA, and BbF) indicates the PAH content of food products. In August 2011, the European Union (EU) introduced Regulation EC No. 835/2011 [20], which recommends the maximum permissible limits of 2.0 µg/kg for BaP and 12.0 µg/kg for the sum of PAH4 in smoked meat and smoked meat products. The Chinese National Standard (CNS) GB 2762-2022 also stipulates a maximum limit of 5.0 µg/kg for BaP in smoked meat products. However, no maximum permitted level of PAH4 has been proposed by Chinese regulations (CN limit).

The existing research on PAH4 content in China and other countries has mainly focused on fish [21,22] and dairy products [19,23]. Bogdanović et al. [24] investigated the levels of BaA, Chr, BbF, and BaP and their sum (PAH4) in a total of 180 samples of fish, shellfish, and meat products produced in Croatia. The mean PAH4 level was 1.47 μg/kg for meat and 1.48 μg/kg for shellfish products, with meat products being the major contributors of BaP and PAH4. Duedahl-Olesen et al. [22] reported the quantification of PAH4 using GC-QTOF-MS. For nine malt samples, the sum of PAH4 ranged from <0.36 μg/kg for raw barley to 26 μg/kg for peat-smoked barley. For five smoked fish samples, the sum of PAH4 ranged from <0.34 μg/kg for cold-smoked salmon to 2.2 μg/kg for hot-smoked mackerel. The limited knowledge on the PAH4 content of smoked meat products might be due to the dry and hard texture of smoked meat products and the interference posed by fat and protein during the extraction process.

On the other hand, formaldehyde is mainly generated under anaerobic conditions during the smoking process. Methanol formed by dry distillation is further oxidized to form formaldehyde, which is then adsorbed onto the surfaces of food and contaminates the products [25]. The toxicity of formaldehyde primarily affects the eyes and upper respiratory tract and can concurrently induce carcinogenesis, teratogenesis, and mutagenesis [26]. The IARC classifies formaldehyde as a human carcinogen, with inhalation being the primary route of exposure. Ingested formaldehyde mainly affects the tissues or organs is first comes into contact with. Because formaldehyde is rapidly converted to formic acid, it can damage the mucous membrane of the stomach, leading to hyperkeratosis and gastric ulcers [27]. Current detection methods for formaldehyde are mainly used to determine formaldehyde levels in indoor air [28], aquatic products [29,30], and certain food products, such as shiitake mushrooms [31]. Furthermore, there are limited methods available for the measurement of formaldehyde in smoked meat products, as formaldehyde extraction from such products is more complex and difficult due to incomplete extraction or cloudy extracts.

Food is the main route of exposure to PAHs and formaldehyde [27,32].The regulation of PAH4 and formaldehyde in smoked meat products is relatively lenient in both domestic and foreign markets. EU standards stipulate that the PAH4 level in food products must not exceed 12.0 µg/kg [20]. The European Food Safety Authority recommends that the daily oral intake of formaldehyde not exceed 11 mg/kg [33]. The US EPA has established a maximum daily dose reference (RfD) of 0.2 mg/kg body weight per day for formaldehyde [34]. China has yet to set a limit on the formaldehyde content of food products and has merely set a maximum limit of 10.0 mg/kg on formaldehyde concentration in industrial standards for agriculture according to the NY/T1712-2018 standard for green food - dried aquatic products. However, in reality, the PAH4 content of many marketed products exceeds the stipulated limit, which causes PAH4 and formaldehyde contamination.

PAH4 content is mainly measured using gas chromatography–mass spectrometry (GC–MS) [35,36,37,38] and HPLC–fluorescence detection (HPLC–FLD) [39,40]. HPLC offers several advantages, such as high accuracy, high efficiency, low cost, and ease of use. When used in combination with a fluorescence detector and variable excitation and emission wavelengths, it is capable of meeting the measurement requirements of PAH4. Difficulties exist in the measurement of formaldehyde in food products due to the complex sample composition and interference from various components. Since ultraviolet (UV) radiation is weakly absorbed by formaldehyde, the precolumn derivatization technique can be used to introduce a strong UV-absorbing group to improve measurement sensitivity and enhance the chromatographic performance of formaldehyde. 2,4-dinitrophenylhydrazine (DNPH) is commonly used for derivatization in the chromatographic measurement of formaldehyde [41,42]. The resulting derivative, 2,4-dinitrophenylhydrazone, enables simple, rapid, and reliable measurement of formaldehyde content. Therefore, the present study was designed to determine the PAH4 and formaldehyde content of smoked chicken using high-performance liquid chromatography (HPLC) and formaldehyde extraction by leaching followed by ultra-performance liquid chromatography (UPLC), respectively. The determined PAH4 and formaldehyde levels were subsequently analyzed in combination with color and moisture content. The results of this study provide a practical reference for the development of green processing techniques for the production of healthy and safe smoked chicken products.

## 2. Results and Discussion

### 2.1. PAH4 in Smoked Chicken Products

#### 2.1.1. Concentrations of PAH4 in Smoked Chicken Products

**Figure 1:**
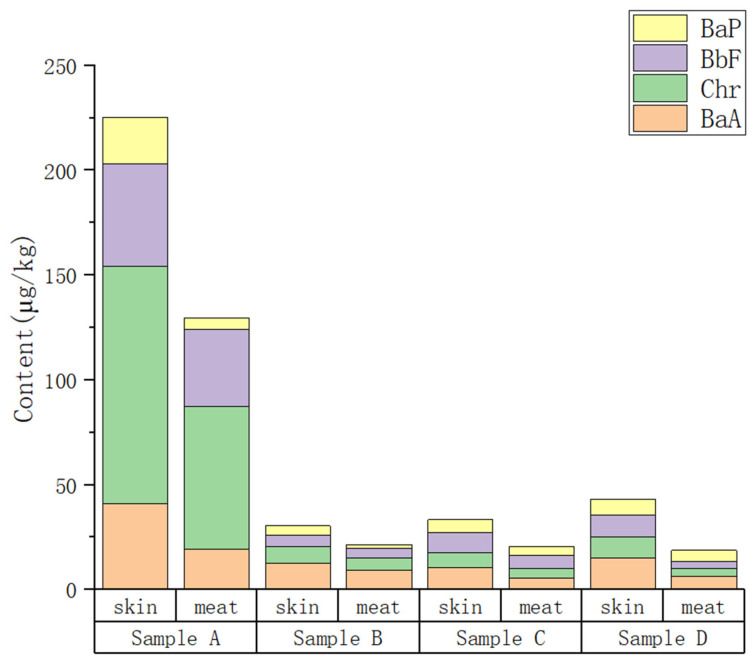
PAH4 content in commercial smoked chicken products.

The PAH4 content of the four smoked chicken products was measured using HPLC. Table 1 and Figure 1 show that all products contained PAH4, and statistically analysis shows that there were significant differences among various products (*p* < 0.05). Among the four commercially available products, with the exception of the BaP content in the lean meat of products A and D, which did not differ significantly (*p* > 0.05), the BaA, Chr, BbF, and BaP content in the smoked skin and meat of product A were significantly different from those of products B, C, and D (*p* < 0.05), with the content in the product A being higher than those of other products. The total PAH4 content in the smoked skin and meat of product A was 225.17 μg/kg and 129.54 μg/kg, respectively, which exceeded the EU regulatory limit. Also, the total PAH4 content in the smoked skin and meat of product B, C, and D exceeded the EU regulations. The content of the four PAHs in the smoked skin and meat of product A, from highest to lowest, was Chr > BbF > BaA > BaP. For product B, the order was BaA > Chr > BbF > BaP. In the smoked skins of product C and D, the contents of the four PAHs were ranked as BaA > BbF > Chr >BaP.

In the four smoked chicken products, the PAH4 content was within the range of 30.43–225.17 µg/kg (average: 83.0 µg/kg) in the smoked skin and 18.75–129.54 µg/kg (average: 48.81 µg/kg) in the smoked lean meat. Except for the BbF content of product B, the content of PAH in the four types of smoked chicken skin was significantly higher than that of lean meat (*p* < 0.05). The significantly higher total PAH4 content of smoked chicken skin compared with meat indicates that smoked chicken skin has a higher level of contamination. This can be explained by the fact that PAH4 contamination in smoked food products originates from the solidification of smoke during the smoking process. Consequently, the PAH4 contaminants are deposited on the surface of food products, with partial migration into the interior of the products. Fasano et al. [19] also pointed to a similar conclusion according to their estimates in cheese and meat sausage: the removal of the external part can be considered a good consumer practice to reduce the ingestion of PAHs.

Wretling et al. [43] reported the measurement of BaP levels in 38 Swedish smoked meats and meat products by means of high-resolution gas chromatography–mass spectrometry (HRGC–MS). Nine samples of smoked meat showed high BaP levels ranging from 6.6 to 36.9 μg/kg, exceeding the 5.0 μg/kg maximum level for smoked meat and fish established by the European Commission (Regulation (EC) No. 208/2005). Our results indicate that the BaP content in the skin and lean meat of traditionally smoked chicken was within the range of 4.33–22.03 μg/kg and 1.84–5.43 μg/kg, respectively. The BaP content in the skin and meat of products A and D and in the skin of product C exceeded the Chinese regulatory limit of 5.0 µg/kg for BaP in smoked meat products. Specifically, the Bap content in the skin and meat of product A was 4.41 times and 1.09 times higher than the Chinese standard limit; the content in the skin and meat of product D was 1.51 times and 1.05 times higher than the standard limit; and the Bap content in the skin of product C was 1.22 times higher than the standard limit. Although the smoked meat products used in the present study and those used by Wretling et al. were from different countries and were subjected to different measurement methods, both studies found excessive levels of BaP in smoked meat when the same maximum limit was adopted for the determination of BaP content. Several studies have been conducted to detect the BaP content of meat products [43,44]. In the present study, we found that the contribution of BaP in the skin to the PAH4 content of the smoked chicken products A to D was within the range of 9.78–18.27%. The contributions of BaP in meat to the PAH4 content of products A, B, C, and D were 4.19%, 6.96%, 20.90% and 27.95%, respectively. This demonstrates that BaP content alone is not a suitable indicator of PAHs in food products [18].

Rozentale et al. [45] reported a total of 128 samples of smoked meat and meat products produced in Latvia that were analyzed using GC-MS/MS to determine the content of four priority polycyclic aromatic hydrocarbons, including BaP, Chr, BaA, and BbF. The median value of PAH4 content (7.96 μg/kg) was higher in smoked chicken samples than in other smoked meat products. In this study, the skin and meat of smoked chicken were separately tested, and results indicate that the PAH4 content of all four commercially available smoked chicken products was higher than the maximum limit stipulated by EU regulations. The ƩPAH4 content in smoked chicken skin was 2.54 to 18.76 times higher than the maximum limit specified by the EU (12.0 µg/kg), and the ƩPAH4 content in smoked chicken meat was 1.56 to 10.80 times higher than the specified limit. This indicates the high level of PAH4 content in traditional Chinese smoked chicken; it is thus necessary to develop new and green processing technology for traditionally smoked meat products due to the hazards of PAH4 to humans [11,12,13,14,15].

#### 2.1.2. Margin of Exposure (MOE) of BaP and ƩPAH4 in Smoked Chicken Products

Table 2 shows the daily exposure of BaP and ƩPAH4 for different populations consuming smoked chicken products. Daily exposure to BaP in the meat and skin of smoked chicken products ranged from 1.37 to 10.99 ng/kg·day and 3.23 to 44.58 ng/kg·day, respectively. In contrast, the daily exposure of ƩPAH4 in the meat and skin of smoked chicken products ranged from 2.06 to 23.73 ng/kg·day and 4.49 to 65.05 ng/kg·day, respectively. Accordingly, children’s BaP and ƩPAH4 exposure was more than 1.8 times higher than the rest of the population. Exposure to BaP and PAH4 was much lower when consuming chicken meat after the removal of the smoked chicken skin.

The risk of consuming BaP and ƩPAH4 via the dietary intake of smoked chicken products was characterized by calculating MOEs (Table 3). An MOE of 10,000 or higher in general would be interpreted as low concern for public health. At an MOE of <10,000, the consumer’s health may be affected.

Except for children, the MOEs of BaP in meat for the general population in this study were greater than 10,000, indicating that at this dietary exposure level (50 g/day), BaP has little effect on the health of consumers. It should be noted that there was some ingestion health risk for children ingesting smoked chicken meat and for all populations ingesting smoked chicken skin.

The MOEs of ƩPAH4 in meat were all greater than 10,000, indicating that at this dietary exposure level, ƩPAH4 has little effect on consumer health. In addition, the MOEs of ƩPAH4 in skin were also over 10,000 for all populations except children, representing a low health risk of ingestion.

### 2.2. Formaldehyde in Smoked Chicken Products

Table 4 shows that all products contained formaldehyde, with the concentration being significantly higher in skin as compared to the lean meat (*p* < 0.05). The formaldehyde content in the skin and lean meat of product A was significantly different from those of the other three products (*p* < 0.05), and the formaldehyde content of the skin was significantly different from the formaldehyde content of the meat in all four products (*p* < 0.05). In particular, product B had a higher formaldehyde content compared to the other products, with the content in skin and meat being 6.84 mg/kg and 2.95 mg/kg, respectively. In the four smoked chicken products, formaldehyde content was within the range of 2.17–6.84 mg/kg (average: 3.71 mg/kg) in the skin and 0.86–2.95 mg/kg (average: 1.75 mg/kg) in the lean meat. There are currently no national standards in China that limit the formaldehyde content in meat products. However, considering the carcinogenicity, teratogenicity, and mutagenicity of formaldehyde, the content of formaldehyde in food products should be as low as possible.

### 2.3. Moisture Content in Smoked Chicken Products

As shown in Table 5, the moisture content of the four smoked chicken products was within the range of 53.17–67.28% (average: 62.49%). Product A had the lowest moisture content, which was significantly different from that of other products (*p* < 0.05). Product A had a dry and tough texture, dark surface color, and low moisture content, which may be due to a longer smoking time. The low moisture content resulted in poor sensory qualities, a hard texture, and an unpleasant flavor when consumed. Products B, C, and D did not differ significantly in moisture content, and the products were juicier.

### 2.4. Smoked Chicken Product Color

By comparing the color difference values of the four commercially available smoked chicken products (see Table 6 below), it was found that there was a significant difference (*p* < 0.05) in the L* values of the various commercially available products, and there was no significant difference (*p* > 0.05) between products B and C. In particular, product D had a higher L* value of 57.59. The a* value did not differ significantly between products C and D (*p* > 0.05) but differed significantly with those of products A and B (*p* < 0.05), with product B having a high a* value and exhibiting a reddish hue. The b* value of product A differed significantly compared with all other products (*p* < 0.05). In particular, product A exhibited a dark appearance, resulting in a significant difference in color values as compared to other commercially available products (*p* < 0.05).

## 3. Materials and Methods

### 3.1. Sample Collection and Sample Preparation

Smoked chicken was purchased from the local market in Liaocheng, Shandong (sample A), Beizhen, Jinzhou, Liaoning (sample B), Beijing (sample C), and Wenzhou, Zhejiang (sample D), respectively.

The skin and lean meat of the smoked chicken were separated and cut into small pieces, and the samples were crushed using a pulverizer, and stored in a clean packaging bag at −20 °C. All the experiments were performed in triplicate (*n* = 3).

### 3.2. Chemicals and Reagents

PAH4 (benz[a]anthracene, chrysene, benzo[b]fluoranthene, and benzo[a]pyrene) standards and the formaldehyde standard were purchased from Anpel Laboratory Technologies Inc. (Shanghai, China). All standards were 100 mg/L in concentration and the purity was ≥99%.

2,4-dinitrophenylhydrazine (DNPH), sodium acetate, acetic acid (analytical grade), and magnesium sulphate (guaranteed reagent) were purchased from Sinopharm Chemical Reagent Co., Ltd. (Shanghai, China). Acetonitrile (ACN) and methanol (HPLC grade) were supplied by Sigma-Aldrich Company (St. Louis, MO, USA), N-hexane (HPLC grade) was obtained from Merck (Darmstadt, Germany), and dichloromethane (DCM) (analytical grade) was purchased from TEDIA Company (Fairfield, OH, USA).

Primary secondary amine (PSA), particle size 40, octadecyl (C18) solid-phase extraction filler (particle size 40), was purchased from Aomi (Shandong) Technology Co., Ltd. (Jinan, China), and diatomaceous earth as a filling material was obtained from Merck (Darmstadt, Germany).

### 3.3. Preparation of Solution

The DNPH solution (0.6 g/L) was prepared by dissolving 300 mg of DNPH in acetonitrile to a constant volume of 500 mL.

The buffer solution (pH = 5) was prepared by dissolving 2.64 mg of sodium acetate in deionized water and adding 1.0 mL of acetic acid, and then the mixture was diluted to 500 mL with deionized water.

Derivative solution was prepared by mixing the buffer solution and DNPH solution at a ratio of 1:1 (*v*:*v*).

### 3.4. Standard Solution Preparation

Mixed PAH4 standard solution at a concentration of 5.0 µg/mL was prepared by mixing 500 μL of the four standard solutions in acetonitrile to a constant volume of 10 mL. The mixed PAH4 standard solution was diluted into mixed standard working solution with a mass concentration of 1, 5, 10, 20, 50, and 100 ng/mL.

Formaldehyde standard working solutions were prepared by taking 20 µL, 50 µL, 100 µL, 200 µL, and 500 µL of formaldehyde standard solutions, diluting them with buffer solution to 5 mL, and then adding DNPH solution to reach a final concentration of 10 mL. Standard working solutions with concentrations of 0.2, 0.5, 1, 2, and 5.5 µg/mL were obtained. After derivatization, the standard curve was obtained via UPLC analysis.

### 3.5. Extraction, Sample Clean-Up and Determination of HPLC for PAH4

#### 3.5.1. Extraction and Sample Clean-Up

The samples for PAH4 measurement were prepared in accordance with the methods described in CNS GB 5009.265 with appropriate modifications. Briefly, 2 g of sample was weighed and placed in a 50 mL centrifuge tube. Thereafter, 1 g of diatomaceous earth and 10 mL of n-hexane were added. The centrifuge tube was vortexed for 30 s, placed in a water bath at 40 °C, sonicated for 30 min, and centrifuged at 5000 r/min for 5 min. The supernatant produced from centrifugation was collected and blown with nitrogen gas. A total of 4 mL of acetonitrile was added, and the mixture was vortexed for 30 s to mix it. After the addition of 900 mg of magnesium sulfate, 100 mg of primary secondary amine (PSA), and 100 mg of octadecyl (C18) packing material, the mixture was vortexed and centrifuged. The supernatant was obtained and blown with nitrogen to a volume of 1 mL, and the resultant solution was collected in a sampling vial before subsequent measurement.

#### 3.5.2. HPLC Instrumentation and Operating Conditions

A high-performance liquid chromatograph (Waters e2695, Waters corporation, Milford, MA, USA) with a fluorescence detector (Waters 2475, Waters corporation, Milford, MA, USA) was used, and separation was performed with a Waters C18 column (250 × 4.6 mm, 5 μm). As shown in Table 7, gradient elution was performed with ACN (A) and water (B) as the mobile phases, at a flow rate of 1.2 mL/min. The wavelength settings of the fluorescence detector are shown in Table 8. The column temperature was 30 °C, and the sample injection volume was 50 μL.

### 3.6. Margin of Exposure for BaP and PAH4 [46]

According to the *Dietary Guidelines for Chinese Residents* (2007 Edition), the daily intake of livestock and poultry meat is 50 to 75 g. The intake level in this study was set at 50 g/day. The body weight of different populations used was in accordance with the results of the China Health and Diet Survey 2002 (Table 2). BaP ingestion exposure was calculated using the following equation:

$$\text{Daily exposure}\;\left[\frac{ng}{day\cdot kg}\right]=\frac{\text{concentration of BaP}\;\left(\frac{ng}{g}\right)\times \text{ daily intake}\;\left(\frac{g}{day}\right)}{\text{body weight}\;(kg)}$$

To calculate the total PAH4 concentration, BaA, Chr, and BbF were estimated as BaP-equivalent concentrations, and the concentrations of BaA, Chr, and BbF were multiplied by 0.1, 0.01, and 0.1, respectively. ƩPAH4 ingestion exposure was calculated using the following equation:

$$\text{Daily exposure}\;\left[\frac{ng}{day\cdot kg}\right]=\frac{\text{concentration of}\;ƩPAH4\left(\frac{ng}{g}\right)\times \;\text{daily intake}\;\left(\frac{g}{day}\right)}{\text{body weight}\;(kg)}$$

To characterize the risk of BaP and ƩPAH4, the MOE was estimated using the following equation:

$$MOE=\frac{{BMDL}_{10}(ng/kg\cdot day)}{\text{The daily exposure}\;(ng/kg\cdot day)}$$

BMDL_10_ value was set by the dose–response analysis for tumor type. The BMDL_10_ for BaP and ƩPAH4 was 70,000 ng/kg·day and 340,000 ng/kg·day.

### 3.7. Extraction, Sample Clean-Up, and Determination of UPLC for Formaldehyde

#### 3.7.1. Extraction and Sample Clean-Up

The processing and distillation of formaldehyde in smoked chicken samples were carried out in accordance with the steam distillation extraction method described by Zhu et al. [25]. By following the pretreatment method established by Li et al. [35], the distillate obtained from meat samples was derivatized with DNPH prior to measurement using the following procedure: 1.0 mL of the distillate was obtained and placed in a 5 mL stoppered colorimetric tube. Subsequently, 0.3 mL of DNPH solution was added, and the mixture was placed in a water bath at 60 °C for 60 min. After rapid cooling with running water, 2 mL of DCM was added, and the mixture was sequentially vortexed for 1 min and centrifuged at 5000 r/min for 2 min. The solution in the lower layer resulting from centrifugation was collected, and the supernatant was extracted using 1 mL of –DCM before combining it with the lower layer. The combined solution was subsequently dehydrated in an anhydrous sodium sulfate column and dried in a water bath at 60 °C. After cooling, the residue was dissolved in 1 mL of methanol and filtered through a 0.22 μm filter membrane before use for UPLC analysis.

#### 3.7.2. UPLC Instrumentation and Operating Conditions

An ultra-high-performance liquid chromatograph (Agilent 1290, Agilent Technologies, Waldbronn, Germany) with a UV detector was used. Separation was performed with an Agilent Zorbax Eclipse C18 column (2.1 × 50 mm, 1.8 μm). The mobile phase contained a mixture of methyl and water in a ratio of 55:45 (*v*:*v*). The detector wavelength was 356 nm, and the sample injection volume was 2 μL. Analysis was performed at a flow rate of 0.3 mL/min and column temperature of 40 °C.

PAH4 and formaldehyde measurement methods were validated using Annex F of the CNS GB/T 27404-2008 criterion on quality of laboratories—chemical testing of food. Five-point calibration curves with correlation coefficients ≥ 0.99 were established for the measurement of PAH4 and formaldehyde. The limits of detections (LODs) for PAH4 and formaldehyde were assessed by testing a series of spiked blank samples. The spiked concentration at a signal-to-noise (S/N) ratio ≥ 3 was used as the LOD of the method.

### 3.8. Moisture Content

The moisture content of the samples was measured using the direct drying method as described in CNS GB 5009.3-2016. The moisture content of four commercially available smoked lean chicken meat products was tested by adopting a uniform procedure for sample collection. Three replicates were established for each experimental group.

### 3.9. Color Determination

Color parameters were measured using a colorimeter (CR-400, Konica Minolta, Tokyo, Japan). The aperture opening size was 8 mm, and the light source was D65. The observer angle used was placed perpendicular to the surface of the samples in order to obtain an accurate recording of the values. Five repetitions were performed for each sample, and color changes were described by L*, a*, and b*.

### 3.10. Statistical Analysis

The results were statistically analyzed using the SAS version 8.0 software (SAS Institute, Cary, NC, USA) and presented as mean ± standard deviation. Mean differences were performed using Duncan’s multiple comparison method at *p* = 0.05.

## 4. Conclusions

In the present study, the PAH4 content in smoked meat products was measured via HPLC. The results indicate that the method was stable and provided good measurement effects. The formaldehyde content in the products was measured by extracting formaldehyde from the smoked meat products by leaching and then performing UPLC, which is a simple and rapid method with high accuracy and sensitivity (See Supplementary Materials for details).

BaP, BbF, Chr, and BaA were detected in the skin and meat of various smoked chicken products. The BaP content in the skin and meat of products A and D and in the skin of product C exceeded the Chinese regulatory limit of 5.0 µg/kg. The ƩPAH4 content of all smoked chicken products in this study exceeded the EU regulation of 12.0 µg/kg. The PAH4 content in the smoked skin was significantly higher compared to the underlying meat, indicating that the skin was more prone to contamination due to its greater proximity to the smoking material. The risk of BaP and ƩPAH4 in smoked chicken products according to MOEs indicates that ingestion of smoked chicken skin should be avoided, especially for children. The formaldehyde measurement results of the smoked chicken products show that all four products were contaminated, indicating that the smoking process produced harmful substances. The formaldehyde content in the skin was higher compared to that in the inner meat. This was mainly attributed to the fact that the skin of smoked meat products is close to the smoking material and therefore more susceptible to the adsorption of harmful substances.

In conclusion, the present study found that commercially available smoked chicken products contained PAH4 levels that far exceeded the regulatory limit and were also contaminated with formaldehyde. The smoked meat industry requires a pleasant-tasting, healthy, and safe food product. Furthermore, the results of this study can be used as a practical reference for the development of green processing techniques for smoked chicken products.

## Figures and Tables

**Table 1:** PAH4 content in commercial smoked chicken products.

Sample	BaA (μg/kg)		Chr (μg/kg)		BbF (μg/kg)		BaP (μg/kg)		ƩPAH4 (μg/kg)	
	Skin	Meat	Skin	Meat	Skin	Meat	Skin	Meat	Skin	Meat
A	40.96 ± 0.78 ^Aa^	19.42 ± 0.44 ^Ab^	113.28 ± 7.75 ^Aa^	67.95 ± 1.40 ^Ab^	48.91 ± 0.61 ^Aa^	36.74 ± 0.39 ^Ab^	22.03 ± 0.77 ^Aa^	5.43 ± 0.51 ^Ab^	225.17 ± 6.81 ^Aa^	129.54 ± 1.95 ^Ab^
B	12.42 ± 0.05 ^Ca^	9.05 ± 0.19 ^Bb^	7.91 ± 0.39 ^Ba^	5.85 ± 0.77 ^Bb^	5.77 ± 1.08 ^Ca^	4.68 ± 0.27 ^Ca^	4.33 ± 0.45 ^Da^	1.84 ± 0.63 ^Cb^	30.43 ± 1.97 ^Ca^	26.43 ± 0.60 ^Bb^
C	10.54 ± 0.82 ^Da^	5.49 ± 0.32 ^Cb^	6.92 ± 0.63 ^Ba^	4.75 ± 0.41 ^BCb^	9.73 ± 0.29 ^Ba^	5.99 ± 0.16 ^Bb^	6.08 ± 0.54 ^Ca^	4.29 ± 0.34 ^Bb^	33.27 ± 0.62 ^Ca^	20.53 ± 0.54 ^BCb^
D	15.28 ± 0.37 ^Ba^	6.47 ± 0.08 ^Cb^	9.65 ± 0.09 ^Ba^	3.73 ± 0.54 ^Cb^	10.77 ± 0.42 ^Ba^	3.33 ± 0.33 ^Db^	7.53 ± 1.12 ^Ba^	5.24 ± 0.31 ^Ab^	43.13 ± 1.08 ^Ba^	18.75 ± 0.42 ^Cb^

The values are expressed as mean ± standard deviation. The difference between the different letters on each line is significant, and the difference between the different letters on each column is significant (*p* < 0.05).

**Table 2:** Dietary exposures to BaP and ƩPAH4 in smoked chicken products (intake level: 50 g/day).

Population Age Groups	Average Body Weight (kg)	Daily Exposure to BaP in Meat (ng/kg·day)	Daily Exposure to BaP in Skin (ng/kg·day)	Daily Exposure to ƩPAH4 in Meat (ng/kg·day)	Daily Exposure to ƩPAH4 in Skin (ng/kg·day)
Male children	25.92	3.55~10.47	8.35~42.50	5.32~22.62	11.63~62.02
Male teenagers	50.59	1.82~5.37	4.28~21.77	2.73~11.59	5.96~31.77
Male adults	67.08	1.37~4.05	3.23~16.42	2.06~8.74	4.49~23.96
Male elderly	65.15	1.41~4.17	3.32~16.91	2.12~9.00	4.63~24.67
Female children	24.71	3.72~10.99	8.76~44.58	5.58~23.73	12.20~65.05
Female teenagers	47.71	1.93~5.69	4.54~23.09	2.89~12.29	6.32~33.69
Female adults	57.33	1.60~4.74	3.78~19.21	2.41~10.23	5.26~28.04
Female elderly	55.97	1.64~4.85	3.87~19.68	2.46~10.47	5.39~28.72

**Table 3:** MOEs of BaP and ƩPAH4 in smoked chicken products (intake level: 50 g/day).

Population Age Groups	MOE of BaP in Meat	MOE of BaP in Skin	MOE of ƩPAH4 in Meat	MOE of ƩPAH4 in Skin
Male children	6683~19,722	1647~8381	15,031~63,910	5482~29,235
Male teenagers	13,043~38,492	3215~16,357	29,336~124,542	10,702~57,047
Male adults	17,295~51,039	4263~21,689	38,902~165,049	14,190~75,724
Male elderly	16,797~49,571	4140~21,065	37,778~160,377	13,782~73,434
Female children	6371~18,801	1570~7989	14,328~60,932	5227~27,869
Female teenagers	12,301~36,301	3032~15,426	27,665~117,647	10,092~53,797
Female adults	14,781~43,621	3643~18,536	33,236~141,079	12,126~64,639
Female elderly	14,431~42,586	3557~18,097	32,474~138,211	11,838~63,080

**Table 4:** Formaldehyde content in commercial smoked chicken products.

Formaldehyde	Skin (mg/kg)	Meat (mg/kg)
Sample A	2.34 ± 0.05 ^Ca^	0.86 ± 0.01 ^Db^
Sample B	6.84 ± 0.04 ^Aa^	2.95 ± 0.13 ^Ab^
Sample C	3.48 ± 0.01 ^Ba^	2.25 ± 0.09 ^Bb^
Sample D	2.17 ± 0.02 ^Da^	0.95 ± 0.05 ^Cb^

The values are expressed as mean ± standard deviation. The difference between the different letters on each line is significant, and the difference between the different letters on each column is significant (*p* < 0.05).

**Table 5:** Moisture content in commercial smoked chicken products.

	Moisture Content (%)
Sample A	53.17 ± 0.06 ^b^
Sample B	65.17 ± 0.06 ^a^
Sample C	64.35 ± 0.07 ^a^
Sample D	67.28 ± 0.07 ^a^

The values are expressed as mean ± standard deviation. Different letter in the column shows significant difference (*p* < 0.05).

**Table 6:** Color difference in commercial smoked chicken products.

	L*	a*	b*
Sample A	20.20 ± 0.99 ^c^	3.65 ± 0.53 ^c^	2.26 ± 0.21 ^b^
Sample B	41.92 ± 3.27 ^b^	23.00 ± 0.95 ^a^	29.42 ± 2.49 ^a^
Sample C	44.08 ± 1.59 ^b^	11.06 ± 1.88 ^b^	26.42 ± 3.66 ^a^
Sample D	57.59 ± 1.80 ^a^	11.49 ± 1.16 ^b^	29.42 ± 1.32 ^a^

The values are expressed as mean ± standard deviation. The difference between the different letters on each column is significant (*p* < 0.05).

**Table 7:** Program of gradient elution of mobile phases.

Time (min)	A (%)	B (%)
0	60	40
12	90	10
29	90	10
34	60	40

**Table 8:** Program of detection wavelength of fluorescence detector.

Time (min)	Λex (nm)	Λem (nm)
0	277	330
14.5	277	384
18.4	303	436
19.6	280	410

## Data Availability

All available data are contained within the article.

## Supplementary Materials

The following supporting information can be downloaded at: https://www.mdpi.com/article/10.3390/molecules28135143/s1, Figure S1: HPLC chromatograms of PAH4 standards detected using FLD. Peak 1 is BaA, peak 2 is Chr, peak 3 is BbF, and peak 4 is BaP; Figure S2: UPLC chromatograms of formaldehyde standards detected using UV. The first peak is DNPH, and the second peak is the derivatized product formaldehyde, HCHO-DNPH; Table S1: Linear regression equations of PAH4.
