# Supplementary material for: Determination of Four PAHs and Formaldehyde in Traditionally Smoked Chicken Products
Source: Molecules. 2023 Jun 30;28(13):5143. doi: 10.3390/molecules28135143 (PMC10343697; doi:10.3390/molecules28135143)
Supplement: Supplementary file 1 [file molecules-28-05143-s001.zip › molecules-2432879-supplementary.docx]

**Supplementary Materials:**

Quality Assurance of PAH4

UPLC–FLD is a CNS-accepted method for PAH measurement. The peak areas of the four polycyclic aromatic hydrocarbons (BaA, Chr, BbF, BaP) (Y) were considered as ordinate and the PAH4 mass concentration (ng/mL, X) as the abscissa to draw the standard curves, and the regression linear equations of four polycyclic aromatic hydrocarbons were obtained, as shown in Table S1. The correlation coefficient was 0.9954–0.9999, the linear range was 1–100 ng/mL, and the detection limit was 0.33–0.50 µg/kg. This method met the needs of actual detection. The standard chromatogram of four polycyclic aromatic hydrocarbons (PAH4) is shown in Figure S1.


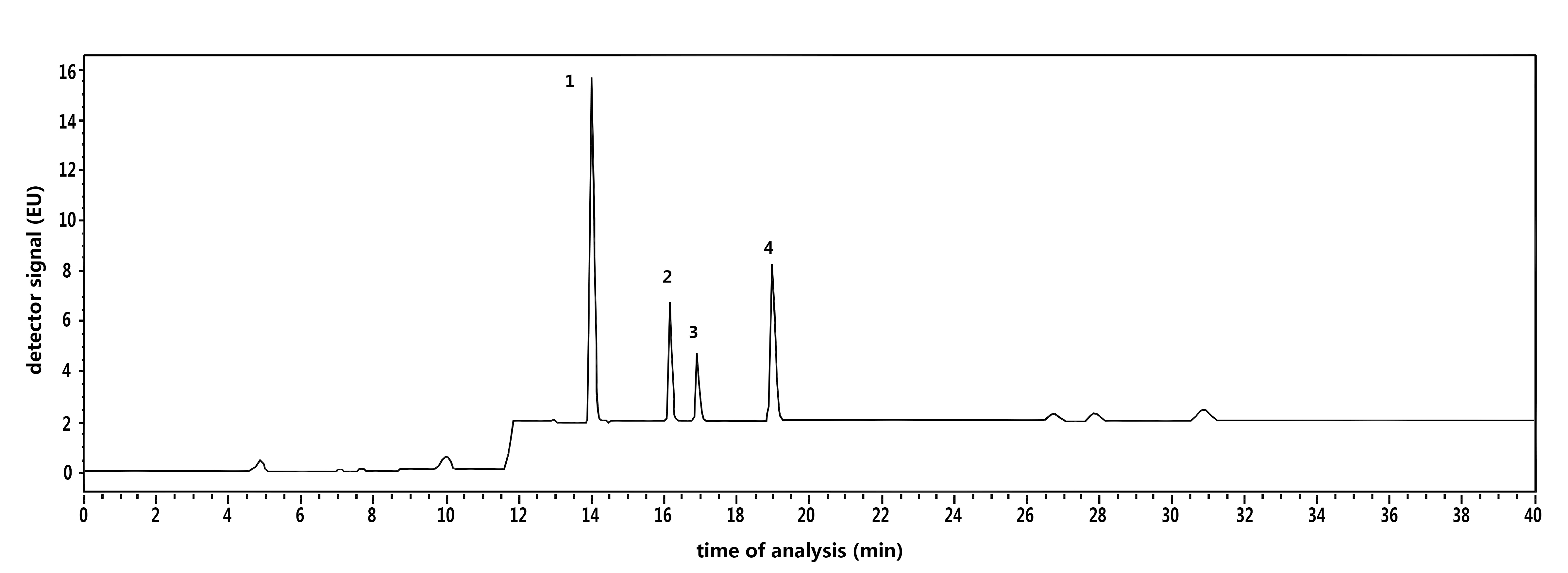


**Figure S1.** HPLC chromatograms of PAH4 standards detected using FLD. Peak 1 is BaA, peak 2 is Chr, peak 3 is BbF, and peak 4 is BaP.

**Table S1.** Linear regression equations of PAH4.

| **PAH4** | **Regression Equation** | **r** | **Linear Range (ng/mL)** |
| --- | --- | --- | --- |
| BaA | y = 11075x + 25214 | 0.9989 | 1~100 |
| Chr | y = 3340.8x − 2010.5 | 0.9999 | 1~100 |
| BbF | y = 2016.3x − 1345.7 | 0.9954 | 1~100 |
| BaP | y = 5637.12x + 3003.6 | 0.9992 | 1~100 |

Quality assurance of formaldehyde.

The standard chromatogram of formaldehyde is shown in Figure S2. The peak area of HCHO-DNPH derivatives (Y) was considered as ordinate and the formaldehyde mass concentration (μg/mL, X) as the abscissa to draw the standard curve. The regression linear equation of formaldehyde was y = 1448.4x − 35.103, its linear correlation coefficient was 0.9956, and the detection limit was 0.17 mg/kg.


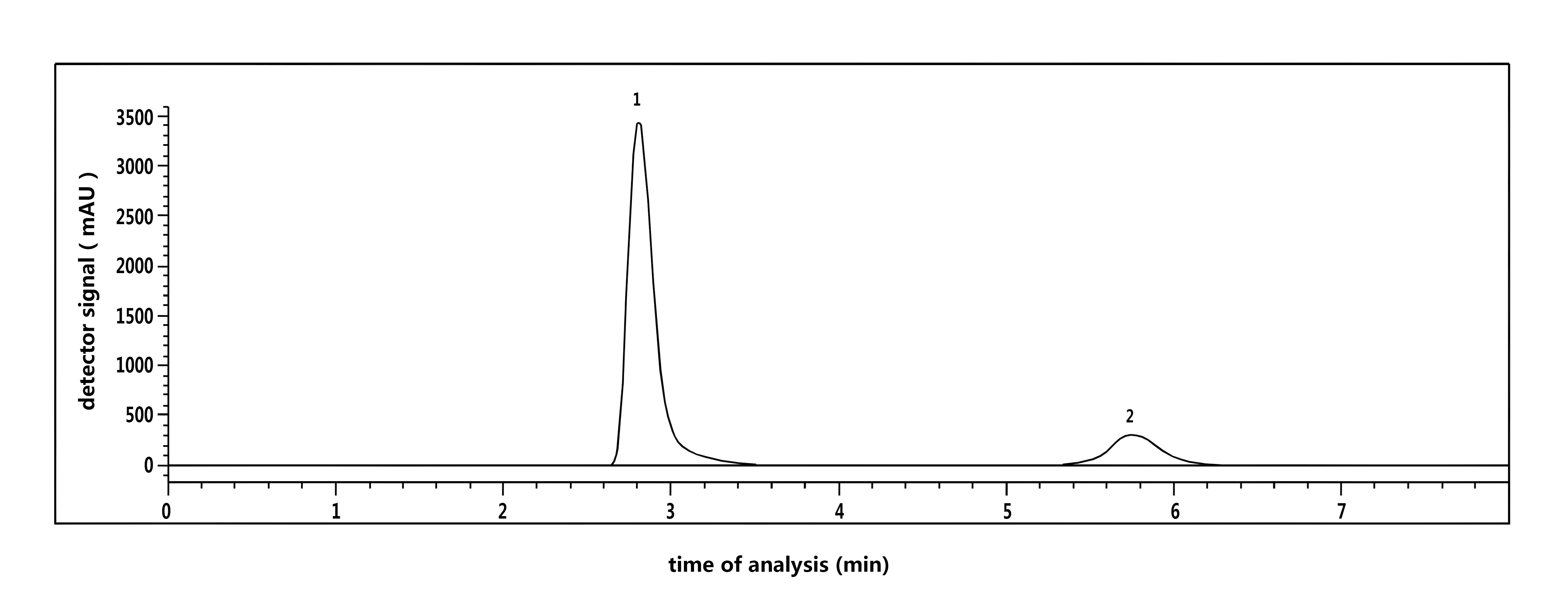


**Figure S2.** UPLC chromatograms of formaldehyde standards detected using UV. The first peak is DNPH, and the second peak is the derivatized product HCHO-DNPH.
